# Specific physical activities, sedentary behaviours and sleep as long-term predictors of accelerometer-measured physical activity in 91,648 adults: a prospective cohort study

**DOI:** 10.1186/s12966-019-0802-9

**Published:** 2019-05-07

**Authors:** Youngwon Kim, Katrien Wijndaele, Stephen J. Sharp, Tessa Strain, Matthew Pearce, Tom White, Nick Wareham, Soren Brage

**Affiliations:** 1 School of Public Health, The University of Hong Kong Li Ka Shing Faculty of Medicine, Room 301D 3/F, Jockey Club Building for Interdisciplinary Research, 5 Sassoon Road, Pokfulam, Hong Kong; 2 MRC Epidemiology Unit, University of Cambridge School of Clinical Medicine, Cambridge, UK

**Keywords:** Physical activity, Sedentary behaviour, Accelerometry, UK biobank, Predictors

## Abstract

**Background:**

The evidence for the prospective relationships between specific physical activities (PA), sedentary behaviours (SB) and sleep on subsequent total PA levels is scarce. The purpose of this study was to examine prospective associations of self-reported PA, SB and sleep, and changes in these with subsequent accelerometer-measured PA.

**Methods:**

A sub-sample of 91,648 UK Biobank participants reported moderate-to-vigorous PA (MVPA), lifestyle activities, TV viewing, computer use and sleep through screen-based questionnaires at baseline (2006–2010), and provided valid accelerometry data (dominant wrist-worn for 7 days between 2013 and 2015). A further sub-sample of 7709 participants repeated the screen-based questionnaires between 2012 and 2013.

**Results:**

In both women (*n* = 51,545) and men (*n* = 40,103), positive associations were observed between all self-reported measures of PA at baseline (MVPA, lifestyle/job-related activities, active transporting modes) and accelerometer-measured PA levels at follow-up (median 5.7 years); an exception was ‘walking/standing at work’ in women. Sedentary time at work, TV viewing and computer use were inversely associated with PA at follow-up. Sleeping either more or less than 7 h/day at baseline was associated with lower PA at follow-up (except for ≤6 h/day in men). In the repeat self-report sub-sample (median 4.3 years), relatively higher physical activity at follow-up was observed in those who maintained or achieved favourable levels of MVPA, walking for pleasure, strenuous sports, other exercises, heavy DIY (in women), heavy physical work, and walking/standing at work (in women), sedentary time at work, getting about methods (in women), commuting methods (in women), TV viewing, computer use or sleep.

**Conclusions:**

Initial levels of PA, SB and sleep, and changes in these variables were generally associated with subsequent accelerometer-measured PA in the expected directions, suggesting these specific behaviours all contribute to the total volume of physical activity over time and could thus be targets for intervention.

**Electronic supplementary material:**

The online version of this article (10.1186/s12966-019-0802-9) contains supplementary material, which is available to authorized users.

## Introduction

Higher overall physical activity (PA) levels are associated with lower risks of cardiometabolic disease [1, 2] and mortality [3]. It is therefore important to understand its determinants. Genetic, biological, behavioural, and environmental factors have all been shown to influence overall PA [4–6]. There are, however, still gaps in our understanding of the behavioural determinants of PA. Associations between prior and future PA levels have been demonstrated, but the quality of the evidence has been criticised, often due to the self-reported nature of outcome PA levels [7]. Cross-sectional associations between sedentary behaviour (SB) and sleep and PA have been examined [8, 9], but there is limited prospective evidence.

The primary aim of this study is, therefore, to investigate the prospective relationship between the self-reported behaviours of sleep, SB and PA in relation to accelerometer-measured PA in the UK Biobank study. The secondary aim is to investigate whether the changes in the self-reported behaviours are prospectively associated with accelerometer-measured PA.

## Methods

### Study design and participants

This study utilises data from the UK Biobank study, an ongoing prospective cohort of over half a million UK adults aged 40–69 years at recruitment. The inclusion criteria were being registered with the National Health Service and residing within < 25 miles of one of 22 assessment centres across the UK. Baseline measurements took place between 2006 and 2010 collecting a wide range of genetic, biological and behavioural information, which included an extensive set of self-reported PA variables, TV viewing, computer use, and sleep. The same set of variables was collected in a repeat-assessment visit (2012–2013) in a subset of individuals. A few years later (2013–2015), 103,711 individuals from the cohort participated in a follow-up study in which they wore an accelerometer on the dominant wrist for 7 days [10]. The methodology of the UK Biobank project and the accelerometry sub-study are described in detail elsewhere [10, 11]. Each participant signed informed consent before participation. The UK Biobank protocol was approved by the North West Multi-Centre Research Ethics Committee.

### Exposures

All behavioural variables were self-reported using touch-screen questionnaires. MVPA time was calculated as the sum of walking time, non-walking moderate (frequency × duration), and vigorous (frequency × duration) activity time performed in a typical week, all of which were reported through questions based on the International Physical Activity Questionnaire [12]. In an additional set of questions, participants were also asked whether they had engaged in any of five specific types of lifestyle activities over the past 4 weeks. Lifestyle activities included walking for pleasure (not as a means of transport), strenuous sports, other exercises (e.g., swimming), light do-it-yourself (DIY) (e.g., pruning) and heavy DIY (e.g., carpentry, digging) activities. Average time spent in each of these activities was obtained by multiplying the reported frequency by the reported average episode duration; all answer options to frequency (e.g., once/4 weeks; 2–3 times/4 weeks; once/week; 2–3 times/week; 4–5 times/week; everyday) and duration (e.g., < 15 min; 15–30 min; 30 min-1 h; 1–1.5 h; 1.5–2 h; 2–3 h; > 3 h) questions were categorical. Participants were also asked about the amount of time spent at work and relative proportions of time spent doing heavy physical work or walking/standing at work. The following weighting scheme was applied to each of four possible choices: 0% for ‘never/rarely’, 25% for ‘sometimes’, 75% for ‘usually’ and 100% for ‘always’. Time spent at work was then multiplied by the weighted relative proportion to obtain estimates of time spent doing ‘Heavy physical work at work’ and ‘Walking/standing at work’. ‘Sedentary time at work’ was estimated as the difference between the total work hours and the sum of ‘Heavy physical work at work’ and ‘Walking/standing at work’. Questions were also provided to obtain information about the participants’ transportation methods to get about (excluding any journeys to and from work), and to commute to and from work. For both getting about and commuting methods, questions included the following four choice categories: car transportation, public transportation, walking, and cycling. Three categories were then defined as follows: car or public transportation (inactive transport), walking or cycling (active transport), and mixed use of inactive and active transport (e.g., car plus cycling or public transport plus walking). TV viewing, computer use time, and sleep (including naps) time on a typical day were each reported in 1-h increments. Sleep was categorised into 5 levels to facilitate the interpretation of the associations with average PA levels: ≤5, 6, 7 (reference), 8 and ≥ 9 h/day. The 7 h/day category was chosen as a reference group, given that sleep time considerably greater or less than about 7 h/day is associated with increased risk of mortality [13, 14].

In a sub-sample of 7709 participants who provided data on these behavioural variables at both baseline and repeat-assessment, three categories of change status were defined using difference values (i.e. repeat-assessment – baseline) for all PA (except transportation methods) and SB variables. For example, individuals were defined as maintainers if the difference between the repeat-assessment and baseline values was within ±5% of the baseline value, decreasers or increasers if the negative difference and positive difference, respectively, was greater than 5%. For both getting about and commuting modes, five categories were defined based on change in the use of transportation modes: 1) ‘Active - Inactive’ (e.g., from walking or cycling at baseline to car or public transport or mixed use at repeat-assessment; from mixed use at baseline to car or public transport at repeat-assessment), 2) ‘Inactive Maintainers’ (e.g., using inactive methods at both baseline and repeat-assessment), 3) ‘Mixed Maintainers’ (e.g., mixed use at both baseline and repeat-assessment), 4) ‘Active Maintainers’ (e.g., using active methods at both baseline and repeat-assessment) and 5) ‘Inactive - Active’ (e.g., from car or public transport at baseline to walking or cycling or mixed use at repeat-assessment; from mixed use at baseline to walking or cycling at repeat-assessment). Nine categories of change were created for sleep: 1) ‘<7 & <7 h/d’ (e.g., less than 7 h/day at both baseline and repeat-assessment), 2) ‘<7 & 7 h/d’ (e.g., less than 7 h/day at baseline but 7 h/day at repeat-assessment), 3) ‘<7 & >7 h/d’ (e.g., less than 7 h/day at baseline but greater than 7 h/day at repeat-assessment), 4) ‘7 & <7 h/d’ (e.g., 7 h/day at baseline but less than 7 h/day at repeat-assessment), 5) ‘7 & 7 h/d’ (e.g., 7 h/day at both baseline and repeat-assessment), 6) ‘7 & >7 h/d’ (e.g., 7 h/day at baseline but greater than 7 h/day at repeat-assessment), 7) ‘>7 & <7 h/d’ (e.g., greater than 7 h/day at baseline but less than 7 h/day at repeat-assessment), 8) ‘>7 & 7 h/d’ (e.g., greater than 7 h/day at baseline but 7 h/day at repeat-assessment), and 9) ‘>7 & >7 h/d’ (e.g., greater than 7 h/day at both baseline and repeat-assessment).

### Physical activity outcome

#### Accelerometer data collection

Invitations to participate in the accelerometer sub-study were sent between 2013 and 2015 to 236,519 participants who had provided an email address at recruitment. Invitations were not sent to individuals in the North West region if they had been involved in other sub-studies due to potentially increased participant burden. Consenting participants received a package which contained an accelerometer (Axivity AX3, Newcastle upon Tyne, UK [15]) initialised to capture three-dimensional acceleration at 100 Hz with a dynamic range of ±8 g, and instructions on proper use of the device. Each participant was asked to begin wearing the accelerometer immediately on their dominant wrist (defined as the hand that participants typically write with) upon receipt for the following seven days. Devices were configured to begin and stop collecting data at pre-specified dates. Participants were asked to return the accelerometer to the co-ordinating centre in a provided pre-paid envelope after the monitoring period ended.

#### Accelerometer data processing

Raw accelerometry data were calibrated to local gravity (1 *g*) with temperature compensation [16] and filtered to dampen machine noise using a fourth-order Butterworth low-pass filter with a cut-off (3 dB) frequency of 20 Hz. Euclidean Norm Minus One (ENMO) was calculated as the Euclidean Norm (vector magnitude) of the acceleration in three axes minus one gravitational unit (1 *g*), with any negative values truncated to zero. Previous research has demonstrated that valid estimates of PA intensity [17] and energy expenditure [18–20] can be obtained from wrist accelerometry. Non-wear was identified as time periods of ≥60 min where standard deviations of all three axes were < 13.0 m-g (1 m-g = 0.001 g) [21]. Overall PA volume (average ENMO) was summarised for each individual activity record, whilst minimising potential diurnal bias caused by non-wear [21]. Participants with < 72 h of wear time or mean ENMO ≥500 m-*g* were excluded from the analysis [22]. For the present analyses, average PA levels were log-transformed, given the non-normality and heteroscedasticity of residuals identified from preliminary analyses.

#### Covariates

We included the following potential confounders of the associations between self-reported behavioural variables and accelerometer-determined PA: age, body mass index (BMI; measured weight in kilograms divided by height in meters squared), ethnicity (White, mixed, Asian/Asian British, Black/Black British, others), smoking status (never, previous, current), employment status (unemployed, employed), time between baseline assessment and accelerometry protocol, accelerometry wear duration (hours/day; obtained from accelerometry data), season of accelerometer wear (two orthogonal sine functions; calculated from timestamp in accelerometry files), severe medical conditions (any of self-reported physician-diagnosed heart attack, stroke or cancer) and grip strength (measured via hand dynamometers).

#### Statistical analyses

Characteristics of men and women at baseline were summarised using means and standard deviations (continuous variables) and percentages (categorical variables). For each baseline behavioural exposure variable, we used linear regression models to estimate the association with subsequent average PA, first with no adjustment for any covariates (Model 1) and second, with covariate adjustment (Model 2). Model 3 was additionally adjusted for the other behaviours under investigation that were not potentially overlapping with the main exposure; for example, the MVPA model 3 was not adjusted for the five specific types of PA. Specific details of the variables included in each model are provided in Additional file 1: Table S1. When the exposure was the change in a behaviour, the models were additionally adjusted for the exposure and any non-overlapping behaviours at baseline. All analyses were stratified by sex. Variance Inflation Factor values of all regression models were less than 10, demonstrating no evidence of severe multi-collinearity. Analyses were performed using Stata/SE Version 14.2 for Windows (StataCorp LLC, College Station, TX).

## Results

A final sample of 51,545 women and 40,103 men with no missing data were included in the analysis (Additional file 1: Figure S1). Table 1 summarises the baseline characteristics of these women and men separately. Additional file 1: Tables S2 and S3 summarise the characteristics of the 7709 participants (4047 women; 3662 men) who attended both the baseline and repeat assessment. In the whole sample, the median time between baseline and accelerometry measurement was 5.7 years (interquartile range: 4.9–6.5 years), and in the subsample the median time between baseline and repeat exposure assessment was 4.3 years (interquartile range: 3.5–4.9 years).

**Table 1:** Baseline characteristics of participants by sex

	Women (*n* = 51,545)	Men (*n* = 40,103)
Age, years	55.6 (7.7)	56.8 (7.9)
Body Mass Index, kg/m^2^	26.2 (4.8)	27.3 (4.0)
Ethnicity		
White	96.9%	97.1%
Non-White	3.2%	2.9%
Smoking status		
Never	60.9%	52.5%
Previous	33.2%	39.4%
Current	5.9%	8.1%
Employment		
Unemployed	38.6%	36.3%
Employed	61.4%	63.7%
Severe medical conditions		
Any of stroke, heart attack or cancer	9.5%	9.5%
Grip strength, kg	24.2 (6.1)	40.3 (8.4)
MVPA, minutes/day	82.7 (95.6)	88.6 (104.0)
Walking for pleasure, minutes/day	15.2 (22.0)	14.5 (21.9)
Strenuous sports, minutes/day	2.0 (8.9)	3.7 (11.7)
Other exercises, minutes/day	9.5 (15.7)	11.0 (19.1)
Light DIY activities, minutes/day	9.7 (21.6)	12.0 (27.1)
Heavy DIY activities, minutes/day	3.8 (11.9)	9.7 (23.3)
Heavy physical work at work, minutes/day	11.4 (33.0)	21.9 (53.3)
Walking/standing at work, minutes/day	43.1 (78.7)	54.3 (90.1)
Sedentary time at work, minutes/day	111.3 (135.8)	137.3 (157.3)
Getting about method		
Car or public transportation	43.5%	43.8%
Mixed use	47.8%	47.0%
Walking or cycling	8.7%	9.3%
Commuting method		
Car or public transportation	85.5%	85.3%
Mixed use	9.4%	10.2%
Walking or cycling	5.0%	4.6%
TV viewing, minutes/day	145.4 (87.7)	149.7 (88.1)
Computer use, minutes/day	65.6 (73.6)	90.0 (88.0)
Sleep, hours/day	7.2 (1.0)	7.1 (1.0)
≤5.0 h/day	3.9%	3.7%
6.0 h/day	17.5%	18.9%
7.0 h/day	41.7%	44.0%
8.0 h/day	30.4%	27.7%
≥9.0 h/day	6.5%	5.8%
Average acceleration, milli-g (at 5.7-year follow-up)	28.5 (8.0)	27.6 (8.8)

Note: Values are means (standard deviations) or percentages, where appropriate. Abbreviations: *DIY* Do-It-Yourself, *MVPA* moderate-to-vigorous physical activity

Tables 2 and 3 show the prospective associations of baseline self-reported behaviours with average accelerometer-measured PA at a median 5.7-year follow-up in women and men, respectively. In general, PA and SB behaviour at baseline had positive and negative associations, respectively, with future overall PA levels (Model 1), which persisted after adjusting for potential confounders (Model 2) and other behaviour variables (Model 3); except for ‘walking/standing at work’ in women which was not significant in Model 3. These associations indicate that accelerometer-measured PA levels were higher in those who at baseline reported spending more time in MVPA, walking for pleasure, strenuous sports, other exercises, light DIY, heavy DIY, heavy physical occupational work, walking/standing at work (only in men), or who used more active getting about or commuting modes at baseline, irrespective of their other behaviours at baseline. Participants who spent less time sedentary at work, watched TV less, or used a computer less at baseline had higher PA levels at follow-up, irrespective of their other baseline behaviours. Compared with the reference category of 7 h/day of sleep, sleeping either more or less than this was associated with lower average accelerometer-measured PA, except for sleeping ≤6 h/day in men.

**Table 2:** Associations of behavioral constructs at baseline with the log of average acceleration levels (milli-g) at follow-up in women

Exposures	Categories	Model 1	Model 2	Model 3
MVPA		0.0005 (0.0004, 0.0005)	0.0004 (0.0004, 0.0004)	0.0004 (0.0004, 0.0004)
Walking for pleasure		0.0014 (0.0013, 0.0015)	0.0014 (0.0013, 0.0015)	0.0011 (0.0010, 0.0012)
Strenuous sports		0.0045 (0.0042, 0.0047)	0.0030 (0.0027, 0.0032)	0.0019 (0.0017, 0.0022)
Other exercises		0.0027 (0.0026, 0.0029)	0.0021 (0.0019, 0.0022)	0.0015 (0.0013, 0.0016)
Light DIY activities		0.0005 (0.0004, 0.0006)	0.0006 (0.0005, 0.0007)	0.0002 (0.0001, 0.0003)
Heavy DIY activities		0.0013 (0.0011, 0.0015)	0.0013 (0.0011, 0.0015)	0.0008 (0.0006, 0.0009)
Heavy physical work at work		0.0008 (0.00077, 0.00092)	0.0005 (0.00047, 0.00060)	0.00050 (0.00039, 0.00061)
Walking/standing at work		0.0004 (0.00039, 0.00045)	0.0002 (0.00019, 0.00026)	−0.0001 (−0.00005, 0.0003)
Sedentary time at work		0.0001 (0.00008, 0.00012)	−0.0002 (− 0.00026, − 0.00022)	−0.0002 (− 0.00026, − 0.00020)
Getting about method	Car or public transportation	(Reference)	(Reference)	(Reference)
	Mixed use	0.0379 (0.0328, 0.0428)	0.0256 (0.0211, 0.0303)	0.0071 (0.0025, 0.0117)
	Walking or cycling	0.0911 (0.0822, 0.0999)	0.0540 (0.0458, 0.0622)	0.0221 (0.0140, 0.0303)
Commuting method	Car or public transportation	(Reference)	(Reference)	(Reference)
	Mixed use	0.0876 (0.0794, 0.0958)	0.0305 (0.0227, 0.0383)	0.0249 (0.0173, 0.0325)
	Walking or cycling	0.1156 (0.1047, 0.1265)	0.0532 (0.0430, 0.0635)	0.0345 (0.0243, 0.0446)
TV viewing		−0.0416 (−0.0432, −0.0401)	−0.0204 (− 0.0219, − 0.0188)	−0.0191 (− 0.0206, − 0.0176)
Computer use		−0.0219 (− 0.0238, − 0.0199)	−0.0176 (− 0.0194, − 0.0159)	−0.0172 (− 0.0190, − 0.0154)
Sleep	≤5.0 h/day	− 0.0627 (− 0.0754, − 0.0501)	− 0.0156 (− 0.0272, − 0.0040)	−0.0134 (− 0.0247, − 0.0019)
	6.0 h/day	− 0.0280 (− 0.0348, − 0.0212)	−0.0082 (− 0.0144, − 0.0020)	−0.0063 (− 0.0124, − 0.0002)
	7.0 h/day	(Reference)	(Reference)	(Reference)
	8.0 h/day	− 0.0264 (− 0.0321, − 0.0207)	−0.0213 (− 0.0265, − 0.0161)	−0.0210 (− 0.0261, − 0.0159)
	≥9.0 h/day	− 0.1051 (− 0.1152, − 0.0951)	−0.0738 (− 0.0831, − 0.0645)	−0.0688 (− 0.0779, − 0.0597)

Model 1: No adjustment

Model 2: Adjustment for age, body mass index, ethnicity, smoking status, employment status, differences in time between baseline and accelerometry protocol, accelerometry wear time, season of accelerometer wear (two orthogonal sine functions), severe medical conditions and grip strength

Model 3: Adjustment for all covariates included in Model 2 plus mutual adjustment for each other. Information about model adjustment is provided in greater detail in Additional file 1: Table S1

Note: The beta coefficients and 95% confidence intervals (values in parentheses) are reported on a log scale. Abbreviations: *DIY* Do-It-Yourself, *MVPA* moderate-to-vigorous physical activity, *SD* Standard Deviation

**Table 3:** Associations of behavioral constructs at baseline with the log of average acceleration levels (milli-g) at follow-up in men

Exposures	Categories	Model 1	Model 2	Model 3
MVPA		0.0005 (0.00047, 0.00053)	0.0005 (0.00046, 0.00051)	0.0005 (0.00043, 0.00049)
Walking for pleasure		0.0008 (0.0006, 0.0009)	0.0013 (0.0012, 0.0014)	0.0011 (0.0009, 0.0012)
Strenuous sports		0.0046 (0.0043, 0.0048)	0.0031 (0.0029, 0.0034)	0.0023 (0.0020, 0.0025)
Other exercises		0.0025 (0.0023, 0.0026)	0.0021 (0.0019, 0.0022)	0.0015 (0.0013, 0.0016)
Light DIY activities		0.0002 (0.0001, 0.0003)	0.0005 (0.0004, 0.0006)	0.0003 (0.0002, 0.0004)
Heavy DIY activities		0.0006 (0.0005, 0.0008)	0.0009 (0.0007, 0.0010)	0.0005 (0.0004, 0.0007)
Heavy physical work at work		0.0009 (0.00081, 0.00092)	0.0006 (0.00056, 0.00067)	0.0004 (0.00032, 0.00045)
Walking/standing at work		0.0005 (0.00049, 0.00056)	0.0003 (0.00029, 0.00037)	0.0001 (0.00005, 0.00015)
Sedentary time at work		0.00017 (0.00015, 0.00019)	−0.0002 (−0.00026, −0.00022)	− 0.0001 (− 0.00014, − 0.00007)
Getting about method	Car or public transportation	(Reference)	(Reference)	(Reference)
	Mixed use	0.0348 (0.0285, 0.0410)	0.0257 (0.0199, 0.0314)	0.0155 (0.0063, 0.0248)
	Walking or cycling	0.0910 (0.0804, 0.1018)	0.0578 (0.0479, 0.0677)	0.0233 (0.0102, 0.0365)
Commuting method	Car or public transportation	(Reference)	(Reference)	(Reference)
	Mixed use	0.1078 (0.0980, 0.1175)	0.0229 (0.0134, 0.0323)	0.011 (0.001, 0.020)
	Walking or cycling	0.1420 (0.1279, 0.1561)	0.0483 (0.0350, 0.0616)	0.025 (0.012, 0.039)
TV viewing		−0.0406 (−0.0426, −0.0386)	−0.0199 (− 0.0218, − 0.0179)	−0.0206 (− 0.0225, − 0.0187)
Computer use		−0.0257 (− 0.0277, − 0.0237)	−0.0211 (− 0.0229, − 0.0192)	−0.0187 (− 0.0205, − 0.0168)
Sleep	≤5.0 h/day	− 0.0401 (− 0.0562, − 0.0240)	−0.0108 (− 0.0257, 0.0040)	−0.0118 (− 0.0263, 0.0027)
	6.0 h/day	−0.0080 (− 0.0162, 0.0001)	0.0024 (− 0.0051, 0.0099)	0.0022 (− 0.0051, 0.0095)
	7.0 h/day	(Reference)	(Reference)	(Reference)
	8.0 h/day	− 0.0498 (− 0.0569, − 0.0426)	−0.0237 (− 0.0304, − 0.0171)	−0.0239 (− 0.0304, − 0.0175)
	≥9.0 h/day	− 0.1455 (− 0.1585, − 0.1324)	−0.0775 (− 0.0897, − 0.0653)	−0.0724 (− 0.0843, − 0.0605)

Model 1: No adjustment

Model 2: Adjustment for age, body mass index, ethnicity, smoking status, employment status, differences in time between baseline and accelerometry protocol, accelerometry wear time, season of accelerometer wear (two orthogonal sine functions), severe medical conditions and grip strength

Model 3: Adjustment for all covariates included in Model 2 plus mutual adjustment for each other. Information about model adjustment is provided in greater detail in Additional file 1: Table S1

Note: The beta coefficients and 95% confidence intervals (values in parentheses) are reported on a log scale. Abbreviations: *DIY* Do-It-Yourself, *MVPA* moderate-to-vigorous physical activity, *SD* Standard Deviation

**Fig. 1:**
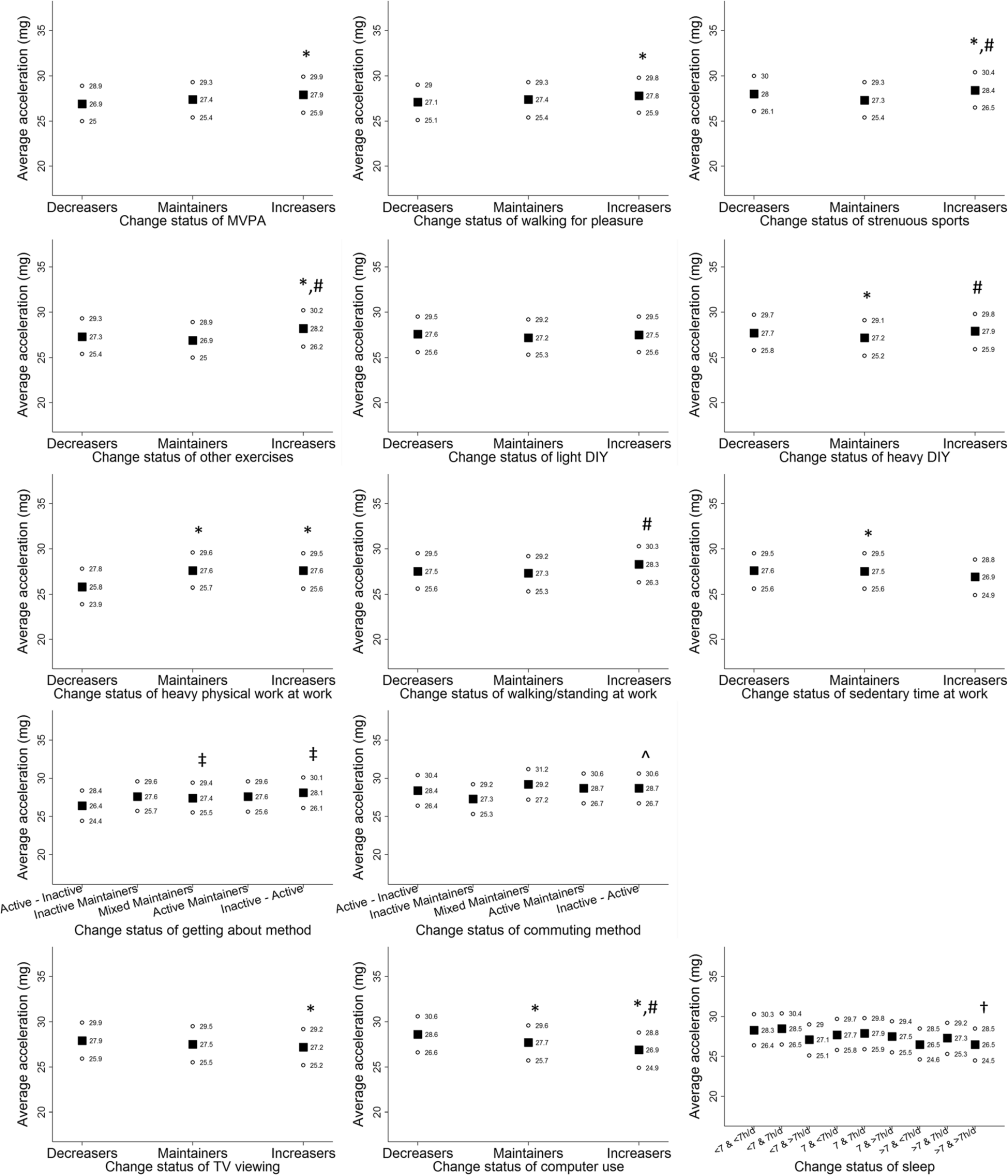
Average acceleration (milli-g) at follow-up (median 5.7 years) within categories of change in each behaviour variable over a median 4.3-year period from baseline to repeat-assessment in women. Note: Values presented are adjusted means of average acceleration obtained from regression models. Information about model adjustment is provided in greater detail in Additional file 1: Table S1. Solid squares and hollow circles represent point estimates and 95% confidence intervals, respectively. “*” for significant differences against ‘Decreasers’; “#” for significant differences against ‘Maintainers; “‡” for significant differences against ‘Active – Inactive’; “^” for significant differences against ‘Inactive Maintainers’; and “†” for significant differences against ‘7 – 7h/d’, at an alpha level of 5%. Abbreviations: DIY – Do-It-Yourself; MVPA – moderate-to-vigorous physical activity

**Fig. 2:**
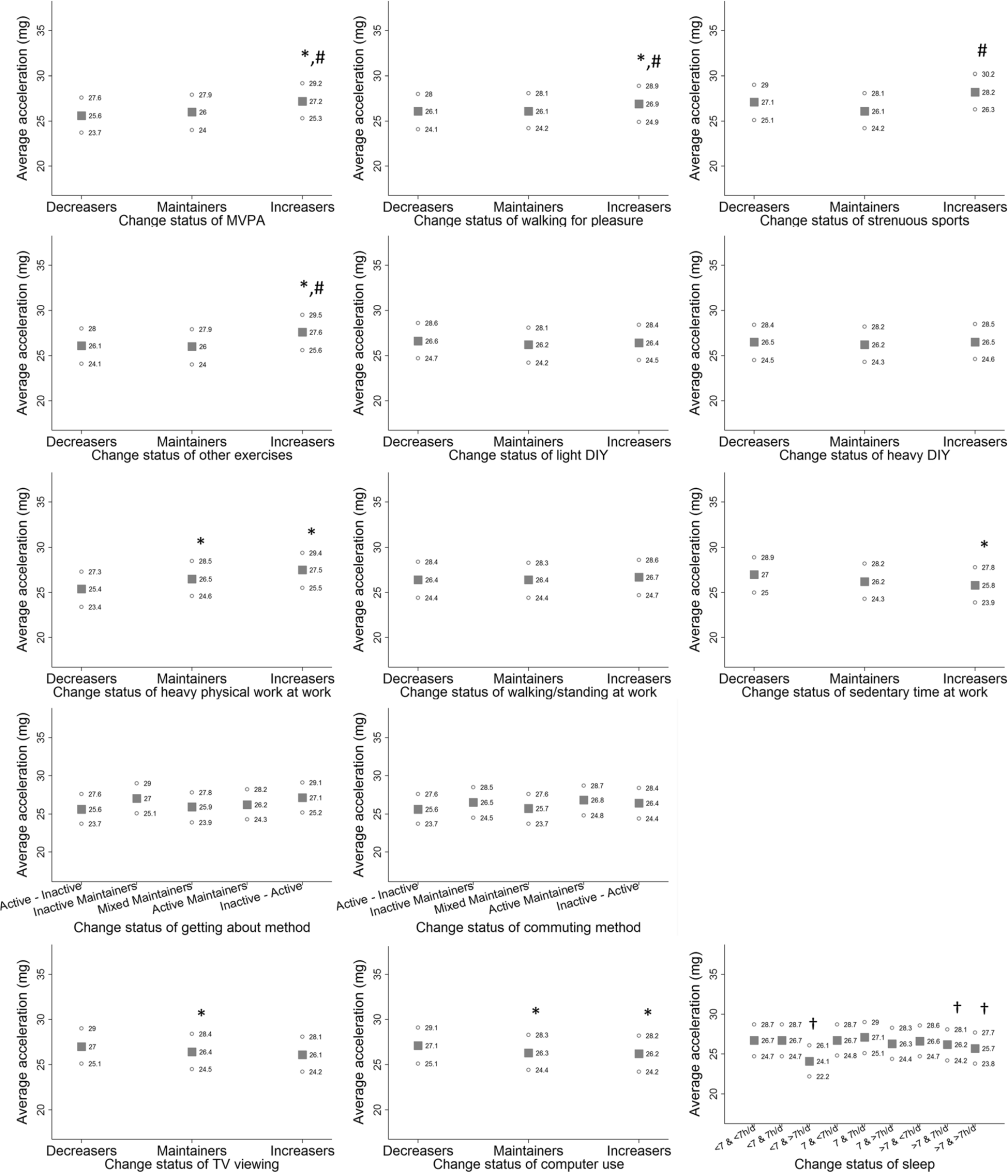
Average acceleration (milli-g) at follow-up (median 5.7 years) according to change status of each behaviour variable over a median 4.3-year period from baseline to repeat-assessment in men. Note: Values presented are adjusted means of average acceleration obtained from regression models. Information about model adjustment is provided in greater detail in Additional file 1: Table S1. Solid squares and hollow circles represent point estimates and 95% confidence intervals, respectively. “*” for significant differences against ‘Decreasers’; “#” for significant differences against ‘Maintainers; “‡” for significant differences against ‘Active – Inactive’; “^” for significant differences against ‘Inactive Maintainers’; and “†” for significant differences against ‘7 – 7h/d’, at an alpha level of 5%. Abbreviations: DIY – Do-It-Yourself; MVPA – moderate-to-vigorous physical activity

Figure 1 shows adjusted mean accelerometer-measured PA levels separately in women who decreased, maintained or increased each self-reported behaviour. Women who maintained or increased time spent in MVPA, walking for pleasure, strenuous sports, other exercises, heavy DIY, heavy physical work and walking/standing at work, active getting about and commuting methods had higher average acceleration levels at follow-up. Accelerometer-measured PA at follow-up was also relatively higher in women who decreased or maintained sedentary time at work, TV viewing time or computer use compared with those who increased time spent in these behaviours. Women whose sleep time was maintained at 7 h/day from baseline to repeat-assessment exhibited marginally higher average acceleration levels at 5.7-year follow-up compared with those who showed other change patterns of sleep: > 7 & > 7 h/d. In men (Fig. 2), maintaining or changing to more favourable status of MVPA, walking for pleasure, strenuous sports, other exercises, heavy physical work at work, sedentary time at work, TV viewing, computer use and sleep was associated with higher subsequent accelerometer-measured PA levels. However, less consistent patterns of associations were observed for change in DIY activities and getting about/commuting methods. The specific point estimates and corresponding 95% confidence intervals used to create Figs. 1 and 2 are provided in Additional file 1: Tables S4 and S5, respectively. In addition, Additional file 1: Table S6 shows associations of change in continuous behavioural variables, with mutual adjustment for the baseline variables.

## Discussion

The present study is the first empirical investigation that provides evidence on the prospective associations of various self-reported behaviours and their changes in relation to subsequent accelerometer-measured PA in a large sample of UK adults. Overall, individuals who had higher PA (including MVPA, lifestyle/occupational activities and active transport), lower SB, or slept 7 h/day at baseline had higher accelerometer-measured PA after a median 5.7-year period of follow-up. In general, individuals who maintained or acquired favourable levels of PA, SB or sleep over a median 4.3-year period had higher accelerometer-measured PA at follow-up.

There have been some studies [23–31] that examined temporal trends of PA, SB or sleep in diverse adult populations using multiple subsequent cross-sectional datasets. These studies [23–31] provide evidence on how the behavioural patterns vary over time, but do not directly address whether these behaviours are predictive of future activity levels. In contrast, results of the present study suggest that women and men are more likely to be physically active in the future not only when they are initially more physically active, less sedentary or sleep approximately 7 h/day, but also when they make desirable changes on these behaviours over time. These findings suggest efforts to reduce sedentary time and maintain approximately 7 h/day of sleep could be additional components to the advice to increase MVPA time in public health policies and interventions that aim to promote physically active lifestyles. These findings will be of interest to other cohort studies that transition to objective measures of PA after baseline measurement(s) using self-report instruments as they have the potential to assist harmonisation work. There are also potentially interesting implications of the finding that prior SB levels are associated with subsequent PA when mutually adjusted for other behaviours.

Another notable observation of this study was the higher accelerometer-measured PA levels at 5.7-year follow-up in individuals whose sleep time was 7 h/day at baseline, or maintained at 7 h/day over a median 4.3-year period. Previous research found that sleeping considerably more or less than 7 h/day (i.e. approximately ≤6 or > 8 h/day) was associated with greater risk of mortality [32], hypertension [33], type 2 diabetes [34], and obesity [35]. Public health efforts are already in place encouraging adults to sleep between 7 and 9 h/day to directly reduce the risk of developing various detrimental health outcomes [13, 14]; our results support these guidelines further by suggesting that they may also promote active living.

The inconsistent results for change status of the DIY, occupational activities, and transportation methods may be attributable to the underlying nature of characterising these behavioural constructs. For example, it may be that subtypes of DIY and occupational activities are so variable that it was particularly difficult for participants not only to quantify specific types of these activities, but also to differentiate between light and heavy DIY activities, and between heavy physical work and walking/standing at work. This may have led to misclassification of such activities at both baseline and repeat-assessment, resulting in potential misclassification of change status of the DIY and occupational activities. With respect to transportation methods, we did not incorporate any information on the frequency and duration of each transportation method (as well as getting about methods), but simply captured the participants’ primary methods of transportation. Although a more systematic approach is needed to investigate change status of DIY, occupational activities and transportation methods relative to future PA, the strong associations of these variables at baseline support their utility as long-term predictors of accelerometer-measured PA.

There are several limitations that need to be considered when interpreting our findings. Firstly, no causal relationships can be inferred due to the observational design of the study. Moreover, the baseline behavioural variables were all assessed through a self-report method, which is associated with measurement error due to recall and social desirability biases. In addition, due to the nature of the questions used in UK Biobank, several behaviours would have been missed and it was also not possible to exclude walking involved in transitioning to cars or public transportation systems in quantification of getting about and commuting methods. There are different limitations to the wrist-acceleration measure of PA including inability to capture all activities and non-representativeness of the week-long monitoring period to tag habitual behaviours; recent methodological studies have shown high validity for this measure in UK adults [19, 20]. Measurement error in exposure variables tend to attenuate the estimated associations, while measurement error in outcome variables widens confidence intervals [36]. Given this, the effect sizes that we report here may have been underestimated due to the potential error in the self-reported exposure variables, but the use of accelerometer-derived PA as an outcome variable should have resulted in smaller standard errors compared with using a self-report measure at follow-up; the latter would also be prone to correlated error which our current analytical design should be less affected by. Furthermore, the findings may not be generalisable to the whole British adult population as no sampling technique was employed to ensure representativeness. Further research on an international scale is needed to examine external validity of our findings in other populations, who differ significantly in behavioural profiles.

## Conclusion

Individuals who reported higher PA (e.g., MVPA, lifestyle/occupational activities, active transport), lower SB (e.g., sedentary time at work, TV viewing, computer use) or slept 7 h/day at baseline were more physically active at 5.7-year follow-up compared with their respective counterparts. In addition, individuals who maintained or achieved more favourable levels of PA, SB or sleep over a 4.3-year period were, in general, more physically active at follow-up. Public health policies and interventions which cause changes in these behaviours may therefore have the potential to promote physically active lifestyles.

## Additional file

Additional file 1: **Table S1.** Schemes for making adjustment for models investigating the associations of baseline variables and change variables with future average acceleration levels. **Table S2.** Baseline characteristics of individuals who provided data at both baseline and repeat-assessment visit. **Table S3.** Characteristics of participants at repeat-assessment visit. **Table S4.** Associations of categorical variables of changes in each behavior between baseline and repeat-assessment visit with the log of average acceleration levels (milli-g). **Table S5.** Associations of categorical variables of changes in each behavior between baseline and repeat-assessment visit with the log of average acceleration levels (milli-g) at follow-up in men. **Table S6.** Associations of continuous variables of changes in each behavior between baseline and repeat-assessment visit with the log of average acceleration levels (milli-g) at follow-up in men. **Figure S1.** Numbers of individuals excluded from and included in the analysis. (DOCX 218 kb)

## Abbreviations

BMI: Body mass index
DIY: Do-It-Yourself
ENMO: Euclidean Norm Minus One
MVPA: Moderate-to-vigorous physical activity
PA: Physical activity
SB: Sedentary behaviour

## References

[1] Wareham NJ (1998). A quantitative analysis of the relationship between habitual energy expenditure, fitness and the metabolic cardiovascular syndrome. Br J Nutr.

[2] Hansen ALS (2013). Combined heart rate- and accelerometer-assessed physical activity energy expenditure and associations with glucose homeostasis markers in a population at high risk of developing diabetes the ADDITION-PRO study. Diabetes Care.

[3] Manini TM (2006). Daily activity energy expenditure and mortality among older adults. Jama-J Am Med Assoc.

[4] Wendel-Vos W (2007). Potential environmental determinants of physical activity in adults: a systematic review. Obes Rev.

[5] Koeneman MA (2011). Determinants of physical activity and exercise in healthy older adults: a systematic review. Int J Behav Nutr Phys Act.

[6] Kirk MA, Rhodes RE (2011). Occupation correlates of adults' participation in leisure-time physical activity: a systematic review. Am J Prev Med.

[7] Condello G (2017). Behavioral determinants of physical activity across the life course: a "DEterminants of DIet and Physical ACtivity" (DEDIPAC) umbrella systematic literature review. Int J Behav Nutr Phys Act.

[8] Mansoubi M (2014). The relationship between sedentary behaviour and physical activity in adults: a systematic review. Prev Med.

[9] Cassidy S (2016). Cross-sectional study of diet, physical activity, television viewing and sleep duration in 233,110 adults from the UK biobank; the behavioural phenotype of cardiovascular disease and type 2 diabetes. BMJ Open.

[10] Doherty A (2017). Large scale population assessment of physical activity using wrist worn accelerometers: the UK biobank study. PLoS One.

[11] UK Biobank Coordinating Centre (2007). UK Biobank: Protocol for a large-scale prospective epidemiological resource. Design.

[12] Craig CL (2003). International physical activity questionnaire: 12-country reliability and validity. Med Sci Sports Exerc.

[13] Kripke DF (2002). Mortality associated with sleep duration and insomnia. Arch Gen Psychiatry.

[14] Ikehara S (2009). Association of sleep duration with mortality from cardiovascular disease and other causes for Japanese men and women: the JACC study. Sleep.

[15] Eijsvogels TMH (2016). Exercise at the extremes the amount of exercise to reduce cardiovascular events. J Am Coll Cardiol.

[16] van Hees VT (2014). Autocalibration of accelerometer data for free-living physical activity assessment using local gravity and temperature: an evaluation on four continents. J Appl Physiol (1985).

[17] Esliger DW (2011). Validation of the GENEA accelerometer. Med Sci Sports Exerc.

[18] Montoye AHK (2016). Wrist-independent energy expenditure prediction models from raw accelerometer data. Physiol Meas.

[19] White T (2016). Estimation of physical activity energy expenditure during free-living from wrist Accelerometry in UK adults. PLoS One.

[20] White, T., et al., Estimating energy expenditure from wrist and thigh accelerometry in free-living adults: a doubly labelled water study. International Journal of Obesity, (in Press). 10.1038/s41366-019-0352-x PMC7358076 30940917

[21] van Hees VT (2011). Estimation of daily energy expenditure in pregnant and non-pregnant women using a wrist-worn tri-axial accelerometer. PLoS One.

[22] Kim Y (2017). Adiposity and grip strength as long-term predictors of objectively measured physical activity in 93,015 adults: the UK biobank study. Int J Obes.

[23] Keadle SK (2016). Prevalence and trends in physical activity among older adults in the United States: a comparison across three national surveys. Prev Med.

[24] Borodulin K (2016). Time trends in physical activity from 1982 to 2012 in Finland. Scand J Med Sci Sports.

[25] Stamatakis E, Ekelund U, Wareham NJ (2007). Temporal trends in physical activity in England: the health survey for England 1991 to 2004. Prev Med.

[26] Knuth AG, Hallel PC (2009). Temporal trends in physical activity: a systematic review. J Phys Act Health.

[27] Juneau CE, Potvin L (2010). Trends in leisure-, transport-, and work-related physical activity in Canada 1994-2005. Prev Med.

[28] Menai M (2014). Changes in sedentary behaviours and associations with physical activity through retirement: a 6-year longitudinal study. PLoS One.

[29] Chau JY (2012). Temporal trends in non-occupational sedentary behaviours from Australian time use surveys 1992, 1997 and 2006. Int J Behav Nutr Phys Act.

[30] Mielke GI (2014). Time trends of physical activity and television viewing time in Brazil: 2006-2012. Int J Behav Nutr Phys Act.

[31] Bin YS, Marshall NS, Glozier N (2012). Secular trends in adult sleep duration: a systematic review. Sleep Med Rev.

[32] Cappuccio FP (2010). Sleep duration and all-cause mortality: a systematic review and meta-analysis of prospective studies. Sleep.

[33] Gangwisch JE (2006). Short sleep duration as a risk factor for hypertension - analyses of the first national health and nutrition examination survey. Hypertension.

[34] Cappuccio FP (2010). Quantity and quality of sleep and incidence of type 2 diabetes: a systematic review and meta-analysis. Diabetes Care.

[35] Cappuccio FP (2008). Meta-analysis of short sleep duration and obesity in children and adults. Sleep.

[36] Hutcheon JA, Chiolero A, Hanley JA (2010). Random measurement error and regression dilution bias. BMJ.

