# Supplementary material for: Specific physical activities, sedentary behaviours and sleep as long-term predictors of accelerometer-measured physical activity in 91,648 adults: a prospective cohort study
Source: Int J Behav Nutr Phys Act. 2019 May 7;16:41. doi: 10.1186/s12966-019-0802-9 (PMC6503547; doi:10.1186/s12966-019-0802-9)
Supplement: Supplementary file 1 — Table S1. Schemes for making adjustment for models investigating the associations of baseline variables and change variables with future average acceleration levels. Table S2. Baseline characteristics of individuals who provided data at both baseline and repeat-assessment visit. Table S3. Characteristics of participants at repeat-assessment visit. Table S4. Associations of categorical variables of changes in each behavior between baseline and repeat-assessment visit with the log of average acceleration levels (milli-g). Table S5. Associations of categorical variables of changes in each behavior between baseline and repeat-assessment visit with the log of average acceleration levels (milli-g) at follow-up in men. Table S6. Associations of continuous variables of changes in each behavior between baseline and repeat-assessment visit with the log of average acceleration levels (milli-g) at follow-up in men. Figure S1. Numbers of individuals excluded from and included in the analysis. (DOCX 218 kb) [file 12966_2019_802_MOESM1_ESM.docx]

Supplementary Table 1. Schemes for making adjustment for models investigating the associations of baseline variables and change variables with future average acceleration levels.

|  | Models examining associations of each exposure variable at baseline | | | Models examining associations of change categories of each exposure variable |
| --- | --- | --- | --- | --- |
| Exposures | Model 1 | Model 2 | Model 3 |  |
| MVPA | No adjustment | Age, body mass index, ethnicity, smoking status, employment status, differences in time between baseline and accelerometry protocol, accelerometry wear time, season of accelerometer wear (two orthogonal sine functions), severe medical conditions and grip strength | All covariates included in Model 2 plus TV viewing, computer use and sleep | All covariates included in Model 2 plus baseline variables of MVPA, TV viewing, computer use, and sleep, and change in TV viewing, computer use and sleep |
| Walking for pleasure |  |  | All covariates included in Model 2 plus strenuous sports, other exercises, light DIY activities, heavy DIY activities, heavy physical work at work, walking/standing at work, sedentary time at work, getting about method, commuting method, TV viewing, computer use and sleep | All covariates included in Model 2 plus baseline variables of walking for pleasure, strenuous sports, other exercises, light DIY, heavy DIY, heavy physical work at work, walking/standing at work, sedentary time at work, getting about methods, commuting methods and sleep, and change status variables of strenuous sports, other exercises, light DIY, heavy DIY, heavy physical work at work, walking/standing at work, sedentary time at work, getting about methods, commuting methods and sleep |
| Strenuous sports |  |  | All covariates included in Model 2 plus walking for pleasure, other exercises, light DIY activities, heavy DIY activities, heavy physical work at work, walking/standing at work, sedentary time at work, getting about method, commuting method, TV viewing, computer use, and sleep | All covariates included in Model 2 plus baseline variables of walking for pleasure, strenuous sports, other exercises, light DIY, heavy DIY, heavy physical work at work, walking/standing at work, sedentary time at work, getting about methods, commuting methods and sleep, and change status variables of other exercises, light DIY, heavy DIY, heavy physical work at work, walking/standing at work, sedentary time at work, getting about methods, commuting methods and sleep |
| Other exercises |  |  | All covariates included in Model 2 plus walking for pleasure, strenuous sports, light DIY activities, heavy DIY activities, heavy physical work at work, walking/standing at work, sedentary time at work, getting about method, commuting method, TV viewing, computer use, and sleep | All covariates included in Model 2 plus baseline variables of walking for pleasure, strenuous sports, other exercises, light DIY, heavy DIY, heavy physical work at work, walking/standing at work, sedentary time at work, getting about methods, commuting methods and sleep, and change status variables of walking for pleasure, strenuous sports, light DIY, heavy DIY, heavy physical work at work, walking/standing at work, sedentary time at work, getting about methods, commuting methods and sleep |
| Light DIY activities |  |  | All covariates included in Model 2 plus walking for pleasure, strenuous sports, other exercises, heavy DIY activities, heavy physical work at work, walking/standing at work, sedentary time at work, getting about method, commuting method, TV viewing, computer use, and sleep | All covariates included in Model 2 plus baseline variables of walking for pleasure, strenuous sports, other exercises, light DIY, heavy DIY, heavy physical work at work, walking/standing at work, sedentary time at work, getting about methods, commuting methods and sleep, and change status variables of walking for pleasure, strenuous sports, other exercises, heavy DIY, heavy physical work at work, walking/standing at work, sedentary time at work, getting about methods, commuting methods and sleep |
| Heavy DIY activities |  |  | All covariates included in Model 2 plus walking for pleasure, strenuous sports, other exercises, light DIY activities, heavy physical work at work, walking/standing at work, sedentary time at work, getting about method, commuting method, TV viewing, computer use, and sleep | All covariates included in Model 2 plus baseline variables of walking for pleasure, strenuous sports, other exercises, light DIY, heavy DIY, heavy physical work at work, walking/standing at work, sedentary time at work, getting about methods, commuting methods and sleep, and change status variables of walking for pleasure, strenuous sports, other exercises, light DIY, heavy physical work at work, walking/standing at work, sedentary time at work, getting about methods, commuting methods and sleep |
| Heavy physical work at work |  |  | All covariates included in Model 2 plus walking for pleasure, strenuous sports, other exercises, light DIY activities, heavy DIY activities, walking/standing at work, sedentary time at work, getting about method, commuting method, TV viewing, computer use, and sleep | All covariates included in Model 2 plus baseline variables of walking for pleasure, strenuous sports, other exercises, light DIY, heavy DIY, heavy physical work at work, walking/standing at work, sedentary time at work, getting about methods, commuting methods and sleep, and change status variables of walking for pleasure, strenuous sports, other exercises, light DIY, heavy DIY, walking/standing at work, sedentary time at work, getting about methods, commuting methods and sleep |
| Walking/standing at work |  |  | All covariates included in Model 2 plus walking for pleasure, strenuous sports, other exercises, light DIY activities, heavy DIY activities, heavy physical work at work, sedentary time at work, getting about method, commuting method, TV viewing, computer use, and sleep | All covariates included in Model 2 plus baseline variables of walking for pleasure, strenuous sports, other exercises, light DIY, heavy DIY, heavy physical work at work, walking/standing at work, sedentary time at work, getting about methods, commuting methods and sleep, and change status variables of walking for pleasure, strenuous sports, other exercises, light DIY, heavy DIY, heavy physical work at work, sedentary time at work, getting about methods, commuting methods and sleep |
| Sedentary time at work |  |  | All covariates included in Model 2 plus walking for pleasure, strenuous sports, other exercises, light DIY activities, heavy DIY activities, heavy physical work at work, walking/standing at work, getting about method, commuting method, TV viewing, computer use, and sleep | All covariates included in Model 2 plus baseline variables of walking for pleasure, strenuous sports, other exercises, light DIY, heavy DIY, heavy physical work at work, walking/standing at work, sedentary time at work, getting about methods, commuting methods and sleep, and change status variables of walking for pleasure, strenuous sports, other exercises, light DIY, heavy DIY, heavy physical work at work, walking/standing at work, getting about methods, commuting methods and sleep |
| Getting about method |  |  | All covariates included in Model 2 plus walking for pleasure, strenuous sports, other exercises, light DIY activities, heavy DIY activities, heavy physical work at work, walking/standing at work, sedentary time at work, commuting method, TV viewing, computer use, and sleep | All covariates included in Model 2 plus baseline variables of walking for pleasure, strenuous sports, other exercises, light DIY, heavy DIY, heavy physical work at work, walking/standing at work, sedentary time at work, getting about methods, commuting methods and sleep, and change status variables of walking for pleasure, strenuous sports, other exercises, light DIY, heavy DIY, heavy physical work at work, walking/standing at work, sedentary time at work, commuting methods and sleep |
| Commuting method |  |  | All covariates included in Model 2 plus walking for pleasure, strenuous sports, other exercises, light DIY activities, heavy DIY activities, heavy physical work at work, walking/standing at work, sedentary time at work, getting about method, TV viewing, computer use, and sleep | All covariates included in Model 2 plus baseline variables of walking for pleasure, strenuous sports, other exercises, light DIY, heavy DIY, heavy physical work at work, walking/standing at work, sedentary time at work, getting about methods, commuting methods and sleep, and change status variables of walking for pleasure, strenuous sports, other exercises, light DIY, heavy DIY, heavy physical work at work, walking/standing at work, sedentary time at work, getting about methods and sleep |
| TV viewing |  |  | All covariates included in Model 2 plus MVPA, computer use and sleep | All covariates included in Model 2 plus baseline variables of MVPA, TV viewing, computer use, and sleep, and change in MVPA, computer use and sleep |
| Computer use |  |  | All covariates included in Model 2 plus MVPA, TV viewing, and sleep | All covariates included in Model 2 plus baseline variables of MVPA, TV viewing, computer use, and sleep, and change in MVPA, TV viewing, and sleep |
| Sleep |  |  | All covariates included in Model 2 plus MVPA, TV viewing and computer use | All covariates included in Model 2 plus baseline variables of MVPA, TV viewing, and computer use, and change in MVPA, TV viewing and computer use |

Supplementary Table 2. Baseline characteristics of individuals who provided data at both baseline and repeat-assessment visit.

|  | Women (n=4,047) | Men (n=3,661) |
| --- | --- | --- |
| Age, years | 56.2 (7.2) | 57.5 (7.5) |
| Body Mass Index, kg/m^2^ | 26.3 (4.8) | 27.2 (3.9) |
| Ethnicity |  |  |
| White | 98.0% | 98.6% |
| Non-White | 2.1% | 1.4% |
| Smoking status |  |  |
| Never | 64.6% | 54.3% |
| Previous | 30.4% | 38.9% |
| Current | 5.0% | 6.8% |
| Employment |  |  |
| Unemployed | 41.6% | 38.4% |
| Employed | 58.4% | 61.6% |
| Severe medical conditions |  |  |
| Any of stroke, heart attack or cancer | 9.1% | 9.5% |
| Grip strength, kg | 25.0 (6.1) | 41.1 (8.4) |
| MVPA, minutes/day | 76.4 (89.3) | 86.4 (100.2) |
| Walking for pleasure, minutes/day | 15.7 (21.6) | 15.6 (22.9) |
| Strenuous sports, minutes/day | 1.8 (8.4) | 3.5 (11.0) |
| Other exercises, minutes/day | 9.0 (14.3) | 11.2 (19.6) |
| Light DIY activities, minutes/day | 10.6 (21.9) | 13.2 (29.0) |
| Heavy DIY activities, minutes/day | 4.0 (11.4) | 10.8 (25.1) |
| Heavy physical work at work, minutes/day | 10.2 (30.7) | 20.4 (51.1) |
| Walking/standing at work, minutes/day | 41.6 (78.6) | 51.3 (84.4) |
| Sedentary time at work, minutes/day | 106.1 (133.9) | 129.4 (153.6) |
| Getting about method |  |  |
| Car or public transportation | 49.0 % | 46.7% |
| Mixed use | 45.3% | 46.0% |
| Walking or cycling | 5.7% | 7.4% |
| Commuting method |  |  |
| Car or public transportation | 89.7% | 89.8% |
| Mixed use | 7.2% | 7.3% |
| Walking or cycling | 3.1% | 2.9% |
| TV viewing, minutes/day | 142.7 (85.3) | 147.9 (86.3) |
| Computer use, minutes/day | 62.8 (71.8) | 84.8 (82.1) |
| Sleep, hours/day | 7.2 (1.0) | 7.2 (0.9) |
| ≤5.0 hours/day | 3.2% | 3.6% |
| 6.0 hours/day | 17.9% | 16.8% |
| 7.0 hours/day | 40.4% | 44.3% |
| 8.0 hours/day | 31.1% | 29.2% |
| ≥9.0 hours/day | 7.4% | 6.2% |
| Average acceleration, milli-g  (at 5.7-year follow-up) | 28.5 (7.9) | 27.6 (8.5) |

Note: Values are means (standard deviations) or percentages, where appropriate. Abbreviations: DIY – Do-It-Yourself; MVPA – moderate-to-vigorous physical activity

Supplementary Table 3. Characteristics of participants at repeat-assessment visit.

|  | Women (n=4,047) | Men (n=3,661) |
| --- | --- | --- |
| Age, years | 60.2 (7.1) | 61.5 (7.3) |
| Body Mass Index, kg/m^2^ | 26.2 (4.8) | 27.3 (4.0) |
| Ethnicity |  |  |
| White | 97.9% | 98.5% |
| Non-White | 2.1% | 1.5% |
| Smoking status |  |  |
| Never | 65.4% | 54.9% |
| Previous | 31.3% | 40.5% |
| Current | 3.3% | 4.7% |
| Employment |  |  |
| Unemployed | 58.8% | 56.6% |
| Employed | 41.2% | 43.4% |
| Severe medical conditions |  |  |
| Any of stroke, heart attack or cancer | 13.2% | 15.1% |
| Grip strength, kg | 19.3 (6.0) | 34.2 (8.2) |
| MVPA, minutes/day | 79.9 (86.9) | 84.1 (96.4) |
| Walking for pleasure, minutes/day | 16.9 (24.1) | 16.9 (24.5) |
| Strenuous sports, minutes/day | 1.9 (9.4) | 3.5 (11.8) |
| Other exercises, minutes/day | 9.7 (15.9) | 10.7 (18.6) |
| Light DIY activities, minutes/day | 8.7 (19.6) | 10.6 (24.2) |
| Heavy DIY activities, minutes/day | 3.1 (10.0) | 8.3 (21.4) |
| Heavy physical work at work, minutes/day | 7.3 (27.1) | 13.9 (42.7) |
| Walking/standing at work, minutes/day | 26.7 (66.7) | 33.3 (71.3) |
| Sedentary time at work, minutes/day | 73.2 (121.7) | 87.4 (139.5) |
| Getting about method |  |  |
| Car or public transportation | 47.6% | 46.1% |
| Mixed use | 45.3% | 46.8% |
| Walking or cycling | 7.1% | 7.1% |
| Commuting method |  |  |
| Car or public transportation | 91.8% | 92.6% |
| Mixed use | 5.8% | 5.1% |
| Walking or cycling | 2.5% | 2.3% |
| TV viewing, minutes/day | 163.9 (92.9) | 166.8 (95.2) |
| Computer use, minutes/day | 81.8 (77.9) | 102.6 (87.4) |
| Sleep, hours/day | 7.2 (1.0) | 7.3 (1.0) |
| ≤5.0 hours/day | 3.9% | 2.8% |
| 6.0 hours/day | 18.0% | 16.7% |
| 7.0 hours/day | 38.6% | 40.3% |
| 8.0 hours/day | 31.5% | 32.2% |
| ≥9.0 hours/day | 7.9% | 8.0% |
| Average acceleration, milli-g  (at 5.7-year follow-up) | 28.5 (7.9) | 27.6 (8.5) |

Note: Values are means (standard deviations) or percentages, where appropriate. Abbreviations: DIY – Do-It-Yourself; MVPA – moderate-to-vigorous physical activity

Supplementary Table 4. Associations of categorical variables of changes in each behavior between baseline and repeat-assessment visit with the log of average acceleration levels (milli-g) at follow-up in women

| Exposure | Change category | Women |  |  |  |
| --- | --- | --- | --- | --- | --- |
| MVPA | Decreasers | (Reference) |  |  |  |
|  | Maintainers | 0.0151 (-0.0189, 0.0492) | (Reference) |  |  |
|  | Increasers | 0.0349 (0.0185, 0.0512) | 0.0197 (-0.0139, 0.0534) |  |  |
|  |  |  |  |  |  |
| Walking for pleasure | Decreasers | (Reference) |  |  |  |
|  | Maintainers | 0.0106 (-0.0108, 0.0320) | (Reference) |  |  |
|  | Increasers | 0.0267 (0.0093, 0.0441) | 0.0161 (-0.0048, 0.0372) |  |  |
|  |  |  |  |  |  |
| Strenuous sports | Decreasers | (Reference) |  |  |  |
|  | Maintainers | -0.0254 (-0.0643, 0.0135) | (Reference) |  |  |
|  | Increasers | 0.0132 (-0.0334, 0.0598) | 0.0836 (0.0059, 0.0714) |  |  |
|  |  |  |  |  |  |
| Other exercises | Decreasers | (Reference) |  |  |  |
|  | Maintainers | -0.0144 (-0.0342, 0.0053) | (Reference) |  |  |
|  | Increasers | 0.0315 (0.0114, 0.0517) | 0.0460 (0.0274, 0.0646) |  |  |
|  |  |  |  |  |  |
| Light DIY activities | Decreasers | (Reference) |  |  |  |
|  | Maintainers | -0.0127 (-0.0327, 0.0073) | (Reference) |  |  |
|  | Increasers | -0.0005 (-0.0194, 0.0182) | 0.0121 (-0.0076, 0.0320) |  |  |
|  |  |  |  |  |  |
| Heavy DIY activities | Decreasers | (Reference) |  |  |  |
|  | Maintainers | -0.0204 (-0.0405, -0.0002) | (Reference) |  |  |
|  | Increasers | 0.0050 (-0.0180, 0.0280) | 0.0254 (0.0054, 0.0454) |  |  |
|  |  |  |  |  |  |
| Heavy physical work at work | Decreasers | (Reference) |  |  |  |
|  | Maintainers | 0.0665 (0.0246, 0.1084) | (Reference) |  |  |
|  | Increasers | 0.0645 (0.0135, 0.1154) | -0.0020 (-0.0427, 0.0386) |  |  |
|  |  |  |  |  |  |
| Walking/standing at work | Decreasers | (Reference) |  |  |  |
|  | Maintainers | -0.0095 (-0.0373, 0.0182) | (Reference) |  |  |
|  | Increasers | 0.0273 (-0.0029, 0.0576) | 0.0369 (0.0072, 0.0665) |  |  |
|  |  |  |  |  |  |
| Sedentary time at work | Decreasers | (Reference) |  |  |  |
|  | Maintainers | -0.0018 (-0.0271, 0.0234) | (Reference) |  |  |
|  | Increasers | -0.0265 (-0.0528, -0.0002) | -0.0247 (-0.0518, 0.0025) |  |  |
|  |  |  |  |  |  |
| Getting about method | Active - Inactive | (Reference) |  |  |  |
|  | Inactive Maintainers | 0.0454 (-0.0028, 0.0937) | (Reference) |  |  |
|  | Mixed Maintainers | 0.0387 (0.0140, 0.0634) | -0.0066 (-0.0530, 0.0396) | (Reference) |  |
|  | Active Maintainers | 0.0443 (-0.0270, 0.1158) | -0.0011 (-0.0874, 0.0852) | 0.0055 (-0.0698, 0.0809) | (Reference) |
|  | Inactive - Active | 0.0626 (0.0194, 0.1058) | 0.0172 (-0.0050, 0.0393) | 0.0238 (-0.0167, 0.0645) | 0.0183 (-0.0653, 0.1019) |
|  |  |  |  |  |  |
| Commuting method | Active - Inactive | (Reference) |  |  |  |
|  | Inactive Maintainers | -0.0412 (-0.1804, 0.0979) | (Reference) |  |  |
|  | Mixed Maintainers | 0.0270 (-0.0307, 0.0848) | 0.0682 (-0.0722, 0.2088) | (Reference) |  |
|  | Active Maintainers | 0.0091 (-0.0807, 0.0989) | 0.0503 (-0.1150, 0.2156) | -0.0179 (-0.1242, 0.0883) | (Reference) |
|  | Inactive - Active | 0.0086 (-0.1237, 0.1410) | 0.0498 (0.0055, 0.0941) | -0.0184 (-0.1518, 0.1149) | -0.0005 (-0.1598, 0.1588) |
|  |  |  |  |  |  |
| TV viewing | Decreasers | (Reference) |  |  |  |
|  | Maintainers | -0.0139 (-0.0375, 0.0097) | (Reference) |  |  |
|  | Increasers | -0.0250 (-0.0494, -0.0006) | -0.0110 (-0.0276, 0.0055) |  |  |
|  |  |  |  |  |  |
| Computer use | Decreasers | (Reference) |  |  |  |
|  | Maintainers | -0.0329 (-0.0580, -0.0077) | (Reference) |  |  |
|  | Increasers | -0.0622 (-0.0876, -0.0368) | -0.0292 (-0.0459, -0.0126) |  |  |
|  |  |  |  |  |  |
| Sleep | <7 & <7h/d | 0.0157 (-0.0102, 0.0415) |  |  |  |
|  | <7 & 7h/d | 0.0209 (-0.0144, 0.0561) |  |  |  |
|  | <7 & >7h/d | -0.0291 (-0.0847, 0.0264) |  |  |  |
|  | 7 & <7h/d | -0.0056 (-0.0391, 0.0279) |  |  |  |
|  | 7 & 7h/d | (Reference) |  |  |  |
|  | 7 & >7h/d | -0.0152 (-0.0431, 0.0128) |  |  |  |
|  | >7 & <7h/d | -0.0500 (-0.1224, 0.0225) |  |  |  |
|  | >7 & 7h/d | -0.0223 (-0.0488, 0.0041) |  |  |  |
|  | >7 & >7h/d | -0.0511 (-0.0722, -0.0300) |  |  |  |

Note: Information about model adjustment is provided in greater detail in Supplementary Table 1. Abbreviations: DIY – Do-It-Yourself; MVPA – moderate-to-vigorous physical activity.

Supplementary Table 5. Associations of categorical variables of changes in each behavior between baseline and repeat-assessment visit with the log of average acceleration levels (milli-g) at follow-up in men

| Exposure | Change category | Men |  |  |  |
| --- | --- | --- | --- | --- | --- |
| MVPA | Decreasers | (Reference) |  |  |  |
|  | Maintainers | 0.0126 (-0.0250, 0.0502) | (Reference) |  |  |
|  | Increasers | 0.0601 (0.0413, 0.0789) | 0.0475 (0.0103, 0.0847) |  |  |
|  |  |  |  |  |  |
| Walking for pleasure | Decreasers | (Reference) |  |  |  |
|  | Maintainers | 0.0022 (-0.0209, 0.0253) | (Reference) |  |  |
|  | Increasers | 0.0309 (0.0109, 0.0509) | 0.0286 (0.0061, 0.0512) |  |  |
|  |  |  |  |  |  |
| Strenuous sports | Decreasers | (Reference) |  |  |  |
|  | Maintainers | -0.0353 (-0.0748, 0.0041) | (Reference) |  |  |
|  | Increasers | 0.0418 (-0.0031, 0.0867) | 0.0771 (0.0456, 0.1087) |  |  |
|  |  |  |  |  |  |
| Other exercises | Decreasers | (Reference) |  |  |  |
|  | Maintainers | -0.0039 (-0.0265, 0.0186) | (Reference) |  |  |
|  | Increasers | 0.05664 (0.0326, 0.0807) | 0.0606 (0.0395, 0.0816) |  |  |
|  |  |  |  |  |  |
| Light DIY activities | Decreasers | (Reference) |  |  |  |
|  | Maintainers | -0.0185 (-0.0412, 0.0042) | (Reference) |  |  |
|  | Increasers | -0.0073 (-0.0291, 0.0145) | 0.0112 (-0.0101, 0.0325) |  |  |
|  |  |  |  |  |  |
| Heavy DIY activities | Decreasers | (Reference) |  |  |  |
|  | Maintainers | -0.0095 (-0.0321, 0.0124) | (Reference) |  |  |
|  | Increasers | 0.0017 (-0.0195, 0.0229) | 0.0112 (-0.0109, 0.0334) |  |  |
|  |  |  |  |  |  |
| Heavy physical work at work | Decreasers | (Reference) |  |  |  |
|  | Maintainers | 0.0449 (0.0048, 0.0850) | (Reference) |  |  |
|  | Increasers | 0.0800 (0.0307, 0.1293) | 0.0351 (-0.0070, 0.0772) |  |  |
|  |  |  |  |  |  |
| Walking/standing at work | Decreasers | (Reference) |  |  |  |
|  | Maintainers | -0.0004 (-0.0322, 0.0314) | (Reference) |  |  |
|  | Increasers | 0.0097 (-0.0229, 0.0432) | 0.0102 (-0.0223, 0.0426) |  |  |
|  |  |  |  |  |  |
| Sedentary time at work | Decreasers | (Reference) |  |  |  |
|  | Maintainers | -0.0282 (-0.0583, 0.0020) | (Reference) |  |  |
|  | Increasers | -0.0434 (-0.0733, -0.0135) | -0.0152 (-0.0496, 0.0192) |  |  |
|  |  |  |  |  |  |
| Getting about method | Active - Inactive | (Reference) |  |  |  |
|  | Inactive Maintainers | 0.0533 (-0.0086, 0.1152) | (Reference) |  |  |
|  | Mixed Maintainers | 0.0093 (-0.0190, 0.0376) | -0.0440 (-0.1031, 0.0151) | (Reference) |  |
|  | Active Maintainers | 0.0227 (-0.0441, 0.0894) | -0.0306 (-0.1209, 0.0597) | 0.0134 (-0.0588, 0.0856) | (Reference) |
|  | Inactive - Active | 0.0564 (-0.0002, 0.1129) | 0.0030 (-0.0227, 0.0287) | 0.0471 (-0.0061, 0.1003) | 0.0337 (-0.0530, 0.1204) |
|  |  |  |  |  |  |
| Commuting method | Active - Inactive | (Reference) |  |  |  |
|  | Inactive Maintainers | 0.0329 (-0.1313, 0.1970) | (Reference) |  |  |
|  | Mixed Maintainers | 0.0016 (-0.0651, 0.0683) | -0.0313 (-0.1994, 0.1369) | (Reference) |  |
|  | Active Maintainers | 0.0434 (-0.0450, 0.1318) | 0.0105 (-0.1756, 0.1966) | 0.0418 (-0.0680, 0.1516) | (Reference) |
|  | Inactive - Active | 0.0294 (-0.1237, 0.1825) | -0.0035 (-0.0654, 0.0584) | 0.0278 (-0.1287, 0.1843) | -0.0140 (-0.1899, 0.1619) |
|  |  |  |  |  |  |
| TV viewing | Decreasers | (Reference) |  |  |  |
|  | Maintainers | -0.0232 (-0.0490, 0.0025) | (Reference) |  |  |
|  | Increasers | -0.0345 (-0.0614, -0.0076) | -0.0113 (-0.0301, 0.0075) |  |  |
|  |  |  |  |  |  |
| Computer use | Decreasers | (Reference) |  |  |  |
|  | Maintainers | -0.0295 (-0.0564, -0.0026) | (Reference) |  |  |
|  | Increasers | -0.0341 (-0.0631, -0.0050) | -0.0046 (-0.0242, 0.0150) |  |  |
|  |  |  |  |  |  |
| Sleep | <7 & <7h/d | -0.0140 (-0.0434, 0.0154) |  |  |  |
|  | <7 & 7h/d | -0.0134 (-0.0499, 0.0230) |  |  |  |
|  | <7 & >7h/d | -0.1155 (-0.1979, -0.0331) |  |  |  |
|  | 7 & <7h/d | -0.0131 (-0.0527, 0.0264) |  |  |  |
|  | 7 & 7h/d | (Reference) |  |  |  |
|  | 7 & >7h/d | -0.0279 (-0.0577, 0.0018) |  |  |  |
|  | >7 & <7h/d | -0.0170 (-0.0924, 0.0583) |  |  |  |
|  | >7 & 7h/d | -0.0339 (-0.0677, -0.0001) |  |  |  |
|  | >7 & >7h/d | -0.0508 (-0.0748, -0.0267) |  |  |  |

Note: Information about model adjustment is provided in greater detail in Supplementary Table 1. Abbreviations: DIY – Do-It-Yourself; MVPA – moderate-to-vigorous physical activity.

Supplementary Table 6. Associations of continuous variables of changes in each behavior between baseline and repeat-assessment visit with the log of average acceleration levels (milli-g) at follow-up in men

|  | **Women** | | | |
| --- | --- | --- | --- | --- |
|  | **Model 2** | | **Model 3** | |
| Exposure | **Baseline^*^** | **Delta^#^** | **Baseline^$^** | **Delta^&^** |
| MVPA^1^ | 0.0007 (0.0006, 0.0008) | 0.0004 (0.0003, 0.0005) | 0.0006 (0.0005, 0.0007) | 0.0004 (0.0003, 0.0005) |
| Walking for pleasure^2^ | 0.0003 (0.0002, 0.0004) | 0.0002 (0.0002, 0.0003) | 0.0002 (0.0002, 0.0003) | 0.0002 (0.0001, 0.0002) |
| Strenuous sports^2^ | 0.0045 (0.0032, 0.0059) | 0.0015 (0.0003, 0.0027) | 0.0031 (0.0017, 0.0044) | 0.0008 (-0.0004, 0.0021) |
| Other exercises^2^ | 0.0031 (0.0024, 0.0038) | 0.0013 (0.0007, 0.0020) | 0.0023 (0.0016, 0.0030) | 0.0010 (0.0004, 0.0016) |
| Light DIY activities^2^ | 0.0008 (0.0003, 0.0012) | 0.0004 (0.000, 0.0008) | 0.0004 (-0.0002, 0.0009) | 0.0003 (-0.0002, 0.0007) |
| Heavy DIY activities^2^ | 0.0016 (0.0006, 0.0025) | 0.0004 (-0.0004, 0.0011) | 0.0005 (-0.0005, 0.0016) | -0.0003 (-0.0011, 0.0005) |
| Heavy physical work at work^2^ | 0.0007 (0.0004, 0.0011) | 0.0005 (0.0001, 0.0009) | 0.0004 (0.0000, 0.0008) | 0.0004 (-0.0001, 0.0008) |
| Walking/standing at work^2^ | 0.0003 (0.0001, 0.0004) | 0.0001 ('0.0000, 0.0003) | 0.0001 (-0.0001, 0.0003) | 0.0001 (-0.0001, 0.0002) |
| Sedentary time at work^2^ | -0.0002 (-0.0003, -0.0001) | -0.0001 (-0.0002, 0.0000) | -0.0002 (-0.0003, -0.0001) | -0.0001 (-0.0002, 0.0000) |
| TV viewing^1^ | -0.0242 (-0.0305, -0.0179) | -0.0127 (-0.0204, -0.0051) | -0.0218 (-0.0279, -0.0158) | -0.0111 (-0.0185, -0.0036) |
| Computer use^1^ | -0.0329 (-0.0403, -0.0254) | -0.0243 (-0.0306, -0.0179) | -0.0314 (-0.0387, -0.0242) | -0.0225 (-0.0287, -0.0163) |
| Sleep 1-5^1^ | 0.0085 (-0.0622, 0.0792) | 0.0289 (-0.0246, 0.0823) | 0.0097 (-0.0600, 0.0795) | 0.0257 (-0.0271, 0.0784) |
| Sleep 6^1^ | 0.0139 (-0.0096, 0.0373) | 0.0025 (-0.0201, 0.0252) | 0.0183 (-0.0046, 0.0412) | 0.0047 (-0.0178, 0.0273) |
| Sleep 7^1^ | Ref | -0.0068 (-0.0239, 0.0103) | Ref | -0.0082 (-0.0247, 0.0084) |
| Sleep 8^1^ | -0.0309 (-0.0499, -0.0118) | -0.0074 (-0.0268, 0.0120) | -0.0319 (-0.0503, -0.0135) | -0.0086 (-0.0275, 0.0103) |
| Sleep 9-18^1^ | -0.1010 (-0.1460, -0.0559) | -0.0140 (-0.0443, 0.0163) | -0.0975 (-0.1399, -0.0551) | -0.0195 (-0.0486, 0.0096) |
| Getting about: Inactive maintainers^2^ | NA | Ref | NA | Ref |
| Getting about:Decreasers^2^ | NA | -0.0461 (-0.0968, 0.0046) | NA | -0.0388 (-0.0876, 0.0099) |
| Getting about: Mixed maintainers^2^ | NA | 0.0104 (-0.0382, 0.0591) | NA | 0.0016 (-0.0451, 0.0483) |
| Getting about: Active maintainers^2^ | NA | 0.0030 (-0.0900, 0.0959) | NA | 0.0002 (-0.0881, 0.0886) |
| Getting about: Increasers^2^ | NA | 0.0332 (0.0101, 0.0563) | NA | 0.0152 (-0.0071, 0.0375) |
| Commuting: Inactive maintainers^2^ | NA | Ref | NA | Ref |
| Commuting: Decreasers^2^ | NA | 0.0468 (-0.1030, 0.1966) | NA | 0.0316 (-0.1121, 0.1752) |
| Commuting: Mixed maintainers^2^ | NA | 0.1065 (-0.0457, 0.2587) | NA | 0.0777 (-0.0679, 0.2233) |
| Commuting: Active maintainers^2^ | NA | 0.0619 (-0.1155, 0.2392) | NA | 0.0462 (-0.1240, 0.2164) |
| Commuting: Increasers^2^ | NA | 0.0741 (0.0261, 0.1221) | NA | 0.0598 (0.0144, 0.1052) |
|  | **Men** | | | |
|  | **Model 1** | | **Model 3** | |
| Exposure | **Baseline^*^** | **Delta^#^** | **Baseline^$^** | **Delta^&^** |
| MVPA^1^ | 0.0008 (0.0007, 0.0009) | 0.0005 (0.0004, 0.0006) | 0.0007 (0.0006, 0.0008) | 0.0005 (0.0004, 0.0006) |
| Walking for pleasure^2^ | 0.0004 (0.0003, 0.0004) | 0.0002 (0.0002, 0.0003) | 0.0003 (0.0002, 0.0004) | 0.0002 (0.0001, 0.0003) |
| Strenuous sports^2^ | 0.0044 (0.0034, 0.0055) | 0.0024 (0.0015, 0.0033) | 0.0030 (0.0018, 0.0042) | 0.0018 (0.0009, 0.0027) |
| Other exercises^2^ | 0.0030 (0.0024, 0.0035) | 0.0019 (0.0013, 0.0024) | 0.0022 (0.0016, 0.0028) | 0.0015 (0.0009, 0.0021) |
| Light DIY activities^2^ | 0.0008 (0.0004, 0.0012) | 0.0002 (-0.0002, 0.0006) | 0.0006 (0.0002, 0.00011) | 0.0002 (-0.0002, 0.0005) |
| Heavy DIY activities^2^ | 0.0009 (0.0004, 0.0014) | 0.0003 (-0.0001, 0.0008) | 0.0004 (-0.0001, 0.0009) | 0.0001 (-0.0004, 0.0005) |
| Heavy physical work at work^2^ | 0.0009 (0.0007, 0.0011) | 0.0008 (0.0005, 0.0010) | 0.0008 (0.0005, 0.0010) | 0.0006 (0.0004, 0.0009) |
| Walking/standing at work^2^ | 0.0005 (0.0003, 0.0006) | 0.0003 (0.0002, 0.0005) | 0.0002 (0.0000, 0.0004) | 0.0001 (0.0000, 0.0003) |
| Sedentary time at work^2^ | -0.0003 (-0.0004, -0.0002) | -0.0002 (-0.0003, -0.0001) | 0.0000 (-0.0001, 0.0001) | -0.0001 (-0.0002, 0.0000) |
| TV viewing^1^ | -0.0281 (-0.0353, -0.0209) | -0.0266 (-0.0361, -0.0171) | -0.0272 (-0.0340, -0.0204) | -0.0243 (-0.0332, -0.0155) |
| Computer use^1^ | -0.0347 (-0.0430, -0.0264) | -0.0184 (-0.0261, -0.0107) | -0.0274 (-0.0356, -0.0192) | -0.0130 (-0.0206, -0.0056) |
| Sleep 1-5^1^ | 0.0080 (-0.0640, 0.0792) | -0.0539 (-0.1208, 0.0131) | 0.0160 (-0.0547, 0.0867) | -0.0458 (-0.1056, 0.0140) |
| Sleep 6^1^ | -0.0090 (-0.0380, 0.0200) | -0.0160 (-0.0476, 0.0155) | -0.0039 (-0.0313, 0.0235) | -0.0101 (-0.0391, 0.0190) |
| Sleep 7^1^ | Ref | -0.0087 (-0.0275, 0.0101) | Ref | -0.0083 (-0.0262, 0.0095) |
| Sleep 8^1^ | -0.0253 (-0.0472, -0.0035) | -0.0148 (-0.0357, 0.0060) | -0.0304 (-0.0512, -0.0094) | -0.0166 (-0.0369, 0.0038) |
| Sleep 9-18^1^ | -0.0917 (-0.1375, -0.0460) | 0.0224 (-0.0257, 0.0705) | -0.0835 (-0.1273, -0.0397) | 0.0249 (-0.0167, 0.0665) |
| Getting about: Inactive maintainers^2^ | NA | Ref | NA | Ref |
| Getting about:Decreasers^2^ | NA | -0.0681 (-0.1353, -0.0009) | NA | -0.0362 (-0.0984, 0.0259) |
| Getting about: Mixed maintainers^2^ | NA | -0.0476 (-0.1123, 0.0169) | NA | -0.0298 (-0.0888, 0.0293) |
| Getting about: Active maintainers^2^ | NA | -0.0370 (-0.1339, 0.0599) | NA | -0.0131 (-0.1038, 0.0776) |
| Getting about: Increasers^2^ | NA | 0.0222 (-0.0049, 0.0492) | NA | 0.0041 (-0.0219, 0.0301) |
| Commuting: Inactive maintainers^2^ | NA | Ref | NA | Ref |
| Commuting: Decreasers^2^ | NA | -0.0317 (-0.1955, 0.1322) | NA | -0.0281 (-0.1858, 0.1295) |
| Commuting: Mixed maintainers^2^ | NA | -0.0336 (-0.2027, 0.1355) | NA | -0.0180 (-0.0888, 0.0293) |
| Commuting: Active maintainers^2^ | NA | 0.0401 (-0.1497, 0.2299) | NA | 0.0553 (-0.1251, 0.2356) |
| Commuting: Increasers^2^ | NA | 0.0216 (-0.0459, 0.0890) | NA | -0.0024 (-0.0648, 0.0600) |

* Adjusted for change in each exposure between baseline and follow up, age, body mass index, ethnicity, smoking status, employment status, differences in time between baseline and accelerometry protocol, accelerometry wear time, season of accelerometer wear (two orthogonal sine functions), severe medical conditions and grip strength

^#^ Adjusted for baseline measurement of each exposure, age, body mass index, ethnicity, smoking status, employment status, differences in time between baseline and accelerometry protocol, accelerometry wear time, season of accelerometer wear (two orthogonal sine functions), severe medical conditions and grip strength

^$^ Adjusted for baseline measurement of all other behaviours in group (see below), changes in exposure for all behaviours in group, age, body mass index, ethnicity, smoking status, employment status, differences in time between baseline and accelerometry protocol, accelerometry wear time, season of accelerometer wear (two orthogonal sine functions), severe medical conditions and grip strength

^&^ Adjusted for changes in exposure for all other behaviours in (see below), baseline measurement of all behaviours in group, age, body mass index, ethnicity, smoking status, employment status, differences in time between baseline and accelerometry protocol, accelerometry wear time, season of accelerometer wear (two orthogonal sine functions), severe medical conditions and grip strength

**^1^** Exposure Group 1

**^2^** Exposure Group 2


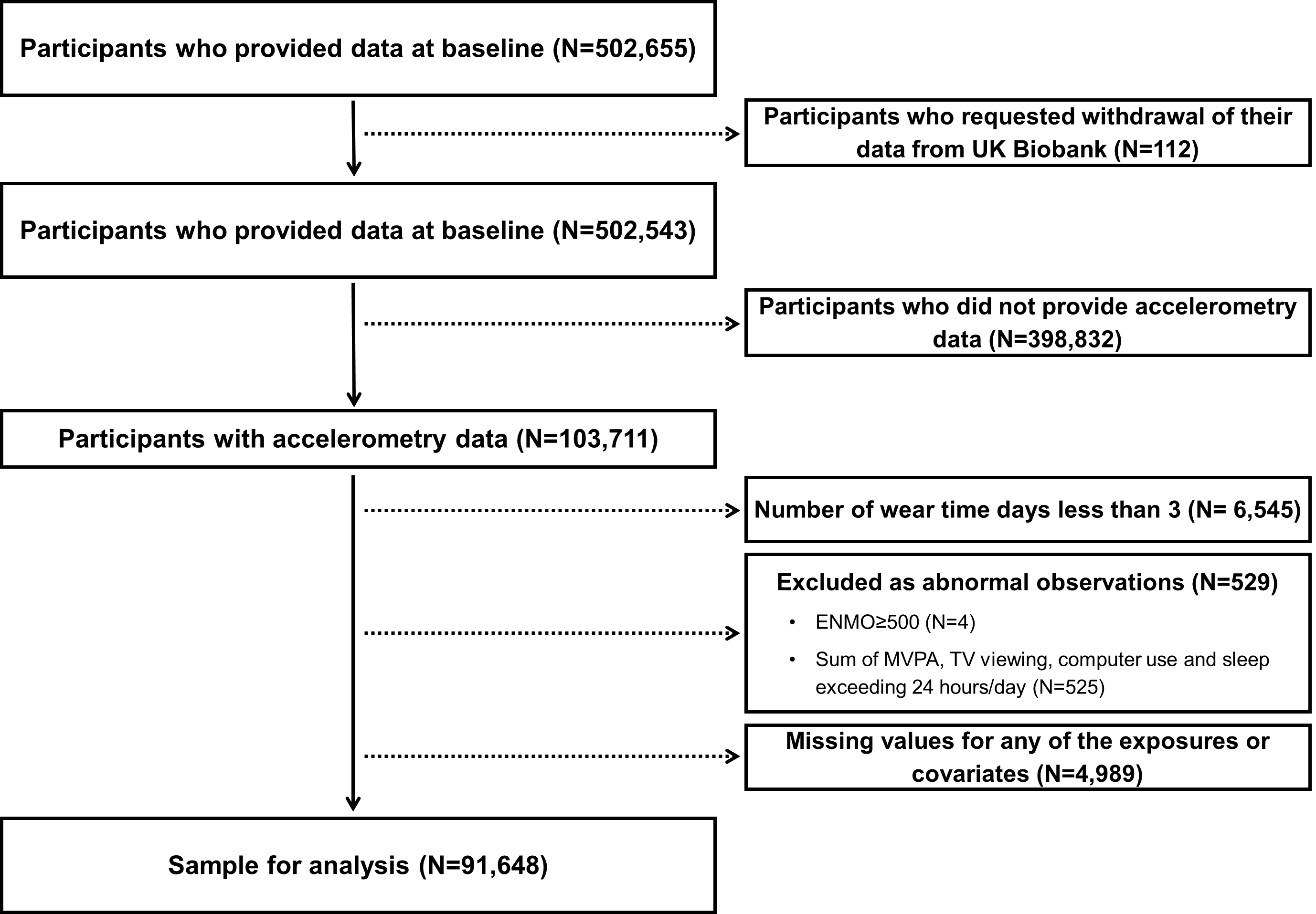


Supplementary Figure 1. Numbers of individuals excluded from and included in the analysis.
